# Food safety: Structure and expression of the asparagine synthetase gene family of wheat

**DOI:** 10.1016/j.jcs.2016.01.010

**Published:** 2016-03

**Authors:** Runhong Gao, Tanya Y. Curtis, Stephen J. Powers, Hongwei Xu, Jianhua Huang, Nigel G. Halford

**Affiliations:** a Biotechnology Research Institute, Shanghai Academy of Agricultural Sciences, 2901 Beidi Road, Minhang District, Shanghai, 201106, PR China; b Plant Biology and Crop Science Department, Rothamsted Research, Harpenden, Hertfordshire AL5 2JQ, United Kingdom; c Computational and Systems Biology Department, Rothamsted Research, Harpenden, Hertfordshire AL5 2JQ, United Kingdom

**Keywords:** Nitrogen metabolism, Nutritional regulation of gene expression, Acrylamide, Crop composition and food safety

## Abstract

Asparagine is an important nitrogen storage and transport molecule, but its accumulation as a free amino acid in crops has implications for food safety because free asparagine is a precursor for acrylamide formation during cooking and processing. Asparagine synthesis occurs by the amidation of aspartate, catalysed by asparagine synthetase, and this study concerned the expression of asparagine synthetase (*TaASN*) genes in wheat. The expression of three genes, *TaASN1-3*, was studied in different tissues and in response to nitrogen and sulphur supply. The expression of *TaASN2* in the embryo and endosperm during mid to late grain development was the highest of any of the genes in any tissue. Both *TaASN1* and *TaASN2* increased in expression through grain development, and in the grain of field-grown plants during mid-development in response to sulphur deprivation. However, only *TaASN1* was affected by nitrogen or sulphur supply in pot-based experiments, showing complex tissue-specific and developmentally-changing responses. A putative N-motif or GCN4-like regulatory motif was found in the promoter of *TaASN1* genes from several cereal species. As the study was completed, a fourth gene, *TaASN4*, was identified from recently available genome data. Phylogenetic analysis showed that other cereal species have similar asparagine synthetase gene families to wheat.

## Highlights

• Three wheat asparagine synthetase genes show differential regulation.
• *TaASN2* is highly expressed specifically in the grain.
• *TaASN2* is a target for reducing the acrylamide-forming potential of wheat.
• A GCN4-like motif is present in the promoter of *TaASN1* genes from multiple species.
• A fourth gene, *TaASN4*, is identifiable from wheat genome data.

## 1 Introduction

Asparagine plays a central role in nitrogen storage and transport in many plant species due to its relatively high ratio of nitrogen to carbon and its relative chemical inertia (Lea et al., 2007). It accumulates in its free (non-protein) form not only during normal physiological processes such as seed germination but also in response to a range of abiotic and biotic stresses (Lea et al., 2007), and understanding the mechanisms that are involved is important for improving crop yield and stress resistance. However, more pressingly, it also has implications for food safety because free asparagine is a precursor for acrylamide formation during high-temperature cooking and processing (reviewed by Curtis et al., 2014a, Halford et al., 2012). This has re-invigorated interest in the enzymes involved in asparagine synthesis and breakdown, and other metabolic pathways that could impact on free asparagine concentrations.

Mineral availability has a major influence on free asparagine accumulation in cereal grain, and highest concentrations occur when the plant has a plentiful supply of nitrogen but is unable to maintain a normal level of protein synthesis because of deficiencies in other nutrients. Unsurprisingly, therefore, nitrogen application correlates positively with free asparagine concentration (Martinek et al., 2009, Postles et al., 2013, Winkler and Schön, 1980), as do deficiencies in potassium, sulphur, phosphorus and magnesium (Lea et al., 2007). In wheat, sulphur deficiency in particular can result in dramatic increases in both the amount of free asparagine in the grain (Curtis et al., 2009, Curtis et al., 2014b, Granvogl et al., 2007, Muttucumaru et al., 2006) and its distribution, with most asparagine accumulating in the embryo when sulphur supply is adequate but with high concentrations occurring in the endosperm under sulphur deficiency (Shewry et al., 2009). This means that wholegrain products are normally more prone to acrylamide formation than white flour products, but that white flour products are disproportionately affected by sulphur deficiency.

Asparagine synthesis occurs by the amidation of aspartate, catalysed by the enzyme asparagine synthetase. Early studies on pea (*Pisum sativum*) identified two, differentially-regulated asparagine synthetase genes (Tsai and Coruzzi, 1990) and two asparagine synthetase genes have already been cloned from wheat (*Triticum aestivum*) and called *TaASN1* and *TaASN2* (Wang et al., 2005). *TaASN1* expression in seedlings was shown to be up-regulated by salt and osmotic stress, and by treatment with abscisic acid (ABA) (Wang et al., 2005). Subsequently, its expression in leaves was shown to be induced by sulphur deficiency, but to be greatly reduced when a general control non-derepressible-2-type protein kinase, TaGCN2, was over-expressed (Byrne et al., 2012).

The study reported here concerned the differential expression of *TaASN1* and *TaASN2*, and a third, hitherto unidentified asparagine synthetase gene, *TaASN3*, in different organs of wheat and under varying levels of nitrogen and sulphur availability. As the study was completed, a fourth gene, *TaASN4*, was identified from recently available genome data.

## 2 Materials and methods

### 2.1 Plant materials and growth conditions

Plant tissues were isolated from wheat (*T. aestivum*) cv. Cadenza. Seeds were surface sterilized in 70% ethanol for 1 min and 10% (v/v) sodium hypochlorite for 20 min with shaking, and then washed thoroughly with distilled water. The sterilized seeds were soaked in distilled water for 6–8 h and then placed in Petri dishes (90 mm) with two layers of moist filter paper at 4 °C for 2 days. The dishes were then transferred to room temperature to allow the seedlings to grow. After 1 week, the seedlings were transplanted to 20 cm diameter pots containing one third sand, one third perlite (typically 70–75% SiO_2_, 12–15% Al_2_O_3_, 3–4% Na_2_O, 3–5% K_2_O, with traces of Fe_2_O_3_, MgO and CaO) and one third nutrient-free compost in a glasshouse with a 16 h day (supplemental lighting was used as necessary) and a minimum temperature of 16 °C. There were five plants per pot.

For the nitrogen feeding study, 24 pots were used, feeding was started one week after transplanting and each pot received 300 mL of liquid every day. Plants were supplied with either a medium containing a full nutrient complement of potassium, phosphate, calcium, magnesium, sodium, iron, nitrate (2 mM Ca(NO_3_)_2_ and 1.6 mM Mg(NO_3_)_2_) and sulphate ions (1.1 mM MgSO_4_) (Curtis et al., 2009, Muttucumaru et al., 2006), or the same medium containing one-tenth the concentration of nitrate (0.2 mM Ca(NO_3_)_2_ and 0.16 mM Mg(NO_3_)_2_). The pots were arranged in a randomised block design with three blocks, giving three replicates of a two treatments by four time points (7, 14, 21 and 28 days post-anthesis) factorial structure. Grain and flag leaf were collected at the four time points (destructive samples from plants in pots), frozen in liquid nitrogen and stored at −70 °C. Embryo and endosperm from grain at 14 days post-anthesis were separated for tissue-specific analysis.

For the nitrogen and sulphur feeding study, seeds were sown and plants were potted up and provided with liquid feed in the same way. This time, 48 pots were used with five plants per pot. The plants were supplied with four different media, each containing a full complement of potassium, phosphate, calcium, magnesium, sodium and iron (Curtis et al., 2009, Muttucumaru et al., 2006) in addition to different concentrations of nitrate and sulphate. These were: 2 mM Ca(NO_3_)_2_, 1.6 mM Mg(NO_3_)_2_ and 1.1 mM MgSO_4_ (the N^+^S^+^ treatment); 0.2 mM Ca(NO_3_)_2_, 0.16 mM Mg(NO_3_)_2_ and 1.1 mM MgSO_4_ (the N–S^+^ treatment); 2 mM Ca(NO_3_)_2_, 1.6 mM Mg(NO_3_)_2_ and 0.11 mM MgSO_4_ (the N^+^S^−^ treatment); 0.2 mM Ca(NO_3_)_2_, 0.16 mM Mg(NO_3_)_2_ and 0.11 mM MgSO_4_ (the N–S^−^ treatment). After four weeks, the concentration of sulphate was reduced to zero in the two sulphur-deficient media. The pots were arranged in a randomised block design with three blocks, giving three replicates of a two nitrogen (N^+^, N^−^) by two sulphur (S^+^, S^−^) by four time points (14, 21, 28, 35 days post-anthesis) factorial structure. Flag leaf, stem, root and grain were collected at 14, 21, 28 and 35 days post-anthesis, and the embryo and endosperm tissues were removed from the grain and separated (samples were not taken at 7 days because the embryo and endosperm are not differentiated at that stage). The material was frozen in liquid nitrogen and kept at −70 °C.

### 2.2 Expression analyses by real-time quantitative polymerase chain reaction (*qPCR*)

RNA was extracted from powdered tissue using the hot phenol method (Verwoerd et al., 1989), with some modification. Approximately 0.5 mL of frozen, powered tissue was suspended in 1 mL of hot (80 °C) phenol and extraction buffer (1:1, v/v). Chloroform:isoamyl alcohol (24:1, v/v) (0.5 mL) was added and mixed well before separation of the phases by centrifugation for 5 min at 4 °C. The aqueous phase was transferred to another tube and the extraction with chloroform:isoamyl alcohol was repeated. Total RNA was then precipitated from the solution by adding lithium chloride to a concentration of 4 M and keeping the solution at 4 °C overnight. After collection of the total RNA by centrifugation and a 70% ethanol wash, any DNA contamination was removed by DNaseI treatment. After further purification by extraction with phenol:chloroform:isoamyl alcohol (25:24:1, v/v/v) and chloroform:isoamyl alcohol, the RNA was precipitated with ethanol and dissolved in diethylpyrocarbonate-treated water. The RNA concentration was measured using a Nanodrop-1000 Spectrophotometer (Thermo Fisher, Wilmington, DE, USA) and its quality checked by electrophoresis through a 1% agarose gel.

RNA samples that had been prepared in the same way were also available from winter wheat, cv. Spark, that had been grown in a field trial on the Rothamsted Research farm at Woburn, Bedfordshire, UK, in 2007–2008. The soil at this site is a sandy loam with very poor nutrient retention and is severely sulphur deficient (extractable soil sulphate concentrations range from 0.5–1.8 mg sulphur per kg; Riley et al., 2002). The plants had been supplied with either 0 (S^−^) or 40 kg sulphur (S^+^) per hectare, in a split plot design, the sulphur being applied as gypsum (calcium sulphate dihydrate) at the tillering stage in March 2008. All of the plants received nitrogen at 200 kg per hectare as ammonium nitrate. There were three replicate samples for each treatment (S^+^, S^−^) at each of four time points (7, 14, 21 and 28 days post-anthesis).

First-strand cDNA synthesis was performed using SuperscriptIII (Invitrogen, Life Technologies Ltd, Paisley, UK) and 2 μg DNase-treated RNA, according to the manufacturer's protocol. The qPCR reaction mix consisted of 10 μL 2 × SYBR Green JumpStart Taq ReadyMix (Sigma, Poole, UK), 2.5 μL cDNA, 1 μL of a 10 μM stock solution of each primer and 0.2 μL ROX reference dye (to normalise the fluorescent reporter signal) in a final volume of 20 μL. The reaction was performed using an Applied Biosystems 7500 Real-Time PCR System (Applied Biosystems, California, USA) and the amplification conditions were 50 °C for 2 min, 95 °C for 10 min, followed by 45 cycles at 95 °C for 15 s and then at 62 °C for 1 min. Data were collected during the 62 °C step and the melting curves were calculated after cycle 45. The primers for qPCR were designed by Primer3 (Untergasser et al., 2012) and are shown in Supplementary Table S1. The specificity of each pair of primers was verified by the amplification of a single band of the expected size, which was checked by gel electrophoresis, nucleotide sequence analysis, and analysis of the dissociation melting curve.

*Ta.2526.1.S1_at*, which encodes a DSS1/SEM1 proteosome, and *TaActin* were used as reference genes. *Ta.2526.1.S1_at* has been shown to have stable expression in developing wheat caryopses (Wan et al., 2013) and *TaActin* is a commonly-used reference gene for qPCR. Reactions were initiated on 96-well plates with two technical replicates per target or reference gene by sample combination. For the nitrogen feeding study seven plates were used: one for each tissue by glasshouse block for grain and flag leaf, and a further plate for embryo and endosperm samples. For the nitrogen and sulphur feeding study 30 plates were used, 10 per glasshouse block of samples. In this case, two technical replicates of a common control sample were also used on each of the plates in the experiment, to consider if there was any untoward plate-to-plate variation. For the study of Spark field samples three plates were used, each accounting for a full set of eight sulphur treatment by time point replicate samples. For ease of processing, genes were randomised on pairs of (the 12) columns and samples on the eight rows. The efficiency of the PCR was estimated using the LinRegPCR programme (Ramakers et al., 2003). A Ct value was obtained by 7500 software v2.0.5 (Applied Biosystems) and the Ct and efficiency values were then used to calculate the Relative Quantity (RQ) and the Normalised Relative Quantity (NRQ) of the target gene's expression with respect to the reference genes. NRQ was calculated using the following formula:

$$\mathrm{NRQ}=\frac{{\bar{E}}_{X}^{-\mathrm{ct,X}}}{\sqrt{{\bar{E}}_{R1}^{-ct,R1}\cdot {\bar{E}}_{R2}^{-\mathrm{ct,R2}}}}$$

where ${\bar{E}}_{X}$ is the average efficiency for a given target gene and where ${\bar{E}}_{R1}$ and ${\bar{E}}_{R2}$ are the average efficiencies for the two reference genes, as calculated from the individual reaction efficiency values provided by LinRegPCR. Following this calculation, a transformation of the NRQ data to log_2_(1/NRQ) (Rieu and Powers, 2009) was used to account for heterogeneity of variance in the NRQ data and so to allow modelling of the data. This provided results on the ct-scale. The method of Residual Maximum Likelihood (REML) was used to fit a linear mixed model to the data. For the nitrogen and sulphur feeding study the model was:

y∼Constant+Tissue∗N∗S∗Time+Block/((Pot/SubSample)+Plate)

where *y* is the transformed NRQ, *Tissue*, *N*, *S* and *Time* are the fixed terms with the asterisk indicating the main effects and interactions between these four factors, and where *Block*, *Pot*, *SubSample* and *Plate* are the random terms (variance components) with the slash indicating the nesting of factors. Here, *Subsample* references the different tissue samples taken from the same *Pot* within a *Block*. Similar models were used for the nitrogen feeding trial and for the Spark grain samples, noting that *Subsample* was not required in the former case as the tissues were analysed on separate plates. In the latter case *Block* was the only random term, which coincided with the plates. For the embryo and endosperm samples from the nitrogen feeding study a t-test was used to compare tissues. Otherwise, the linear mixed modelling analysis tested the main effects and interactions between fixed terms using F-tests while taking account of the variance contributions of the random terms. Significant (p < 0.05, F-test) terms for inspection were disseminated by considering differences between means of biological interest in terms of the standard error of the difference (SED) values on the relevant degrees of freedom (df). The least significant difference (LSD) was therefore used to judge significance at the 5% level.

### 2.3 Genome data mining and nucleotide sequence data analysis

The Genbank nucleotide sequence database and the non-redundant protein database were searched using the National Centre for Biotechnology Information portal (http://www.ncbi.nlm.nih.gov/) and the BLAST tools available therein. Wheat genome data were accessed *via* Cerealsdb (http://www.cerealsdb.uk.net/cerealgenomics/CerealsDB/) (Wilkinson et al., 2012). Amino acid and nucleotide sequence alignments were made using either Geneious version 8 (http://www.geneious.com; Kearse et al., 2012) or the alignment tools provided by EMBL-EBI (http://www.ebi.ac.uk/Tools/psa/). Geneious was also used for phylogenetic analyses.

## 3 Results

### 3.1 Identification of a novel wheat asparagine synthetase gene: TaASN3

Two wheat asparagine synthetase gene sequences had been published previously and called *TaASN1* (Genbank BT009245) and *TaASN2* (Genbank BT009049) (Wang et al., 2005). A BLAST search of the Genbank database using the National Centre for Biotechnology Information portal, *TaASN1* as the query sequence and default search settings, identified a third asparagine synthetase gene, Genebank AK333183, and this was given the name *TaASN3*. *TaASN1*, *TaASN2* and *TaASN3* encode proteins of 586, 582 and 588 amino acids, respectively, with predicted molecular masses of 65.5, 65.1, and 65.8 kDa. An alignment of the predicted protein sequences (Supplementary Fig. S1) revealed that the TaASN1 and TaASN2 proteins shared 87.9% amino acid sequence identity, TaASN1 and TaASN3 76.8% and TaASN2 and TaASN3 74.6%, indicating that TaASN1 and TaASN2 are closely related while TaASN3 is more divergent. All three have the conserved amino acid residues that are known to be essential for aspartate, adenosine triphosphate (ATP) and glutamine binding (Supplementary Fig. S1).

### 3.2 Evidence that asparagine synthetase genes, TaASN1, TaASN2 and TaASN3, are differentially expressed, tissue-specifically, developmentally, and in response to nitrogen and sulphur availability

The fact that free asparagine is a precursor for acrylamide formation during high-temperature cooking and processing makes the expression of asparagine synthetase genes in wheat grain of particular interest. An initial experiment was performed to determine which of the three genes, *TaASN1*, *2* and *3*, was expressed at highest levels in the grain, and whether the genes were differentially regulated through grain development. Grain was sampled at 7, 14, 21 and 28 days post-anthesis, and expression levels of *TaASN1*, *2* and 3 were analysed using quantitative, real-time polymerase chain reaction (qPCR). Given the changes in free asparagine that occur in response to nitrogen availability (Martinek et al., 2009, Postles et al., 2013, Winkler and Schön, 1980), samples for analysis were taken from plants growing in a mixture of perlite, sand and nutrient-free compost and supplied with either a medium containing a full nutrient complement, including 2 mM Ca(NO_3_)_2_ and 1.6 mM Mg(NO_3_)_2_ (Curtis et al., 2009, Muttucumaru et al., 2006), or the same medium containing one-tenth the concentration of nitrate. Two reference genes were used, *Ta.2526.1.S1_at* and *TaActin*, and the data were processed to give results on the ct-scale for statistical analysis. Linear mixed modelling (using REML) was applied to test (F-tests) the main effects and interactions of the two factors of N-treatment and developmental time.

**Fig. 1:**
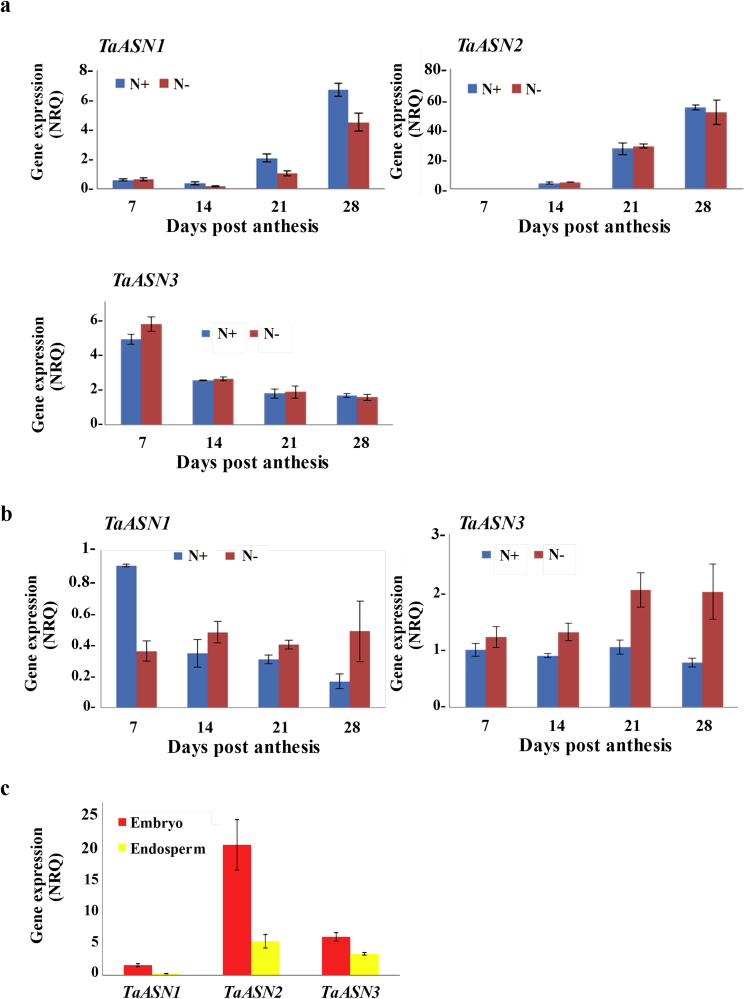
Differential expression of asparagine synthetase genes (*TaASN1*, *TaASN2* and *TaASN3*) in wheat (*Triticum aestivum*) cv. Cadenza developing seeds from plants grown with or without sufficient nitrogen (N+ and N−, respectively). Expression was measured by real-time PCR and the graphs show the NRQ means and standard errors. Statistical analysis of the data is provided in Supplementary Tables S2–S5. a. Relative expression levels of *TaASN1*, *TaASN2* and *TaASN3* in grain at 7, 14, 21 and 28 days post-anthesis. b. Relative expression levels of *TaASN1* and *TaASN3* in flag leaves at 7, 14, 21 and 28 days post-anthesis. c. Relative expression levels of *TaASN1*, *TaASN2* and *TaASN3* in embryo and endosperm at 14 days post-anthesis.

The p-values for the effects of time (days post-anthesis), treatment (N+ versus N-), and the interaction between the two (treatment × time) are given in Supplementary Table S2, and the relevant means, SED (df) and LSD (5%) values for comparisons are given in Supplementary Table S3. The analysis showed the three genes to be differentially regulated, and this is shown graphically in Fig. 1a. *TaASN1* and *TaASN2* showed a similar pattern of expression, increasing from low levels at 7 days post-anthesis to much higher levels at 28 days post-anthesis, but *TaASN2* expression was much higher than that of *TaASN1*, particularly by grain filling. In contrast, *TaASN3* was the most highly expressed of the three genes at 7 days post-anthesis but its expression declined through development. This meant that *TaASN2* was the predominantly expressed gene during the grain filling stage. Neither *TaASN2* nor *TaASN3* were affected significantly by the nitrogen treatment, but *TaASN1* expression did show a significant (p < 0.001, F-test) effect of nitrogen treatment on its own and a borderline significant (p = 0.051, F-test) effect of the treatment interacting with time. Notably, expression during the grain filling phase increased with N feeding (Supplementary Table S3; Fig. 1a).

Expression was also analysed in flag leaves and again a picture of differential expression emerged. *TaASN2* expression was almost undetectable and was not included in the statistical analysis, but the linear mixed modelling of the data for *TaASN1* and *TaASN3* (Supplementary Table S4) showed significant effects of time (p = 0.032, F-test) and treatment × time for *TaASN1*, and a significant effect of treatment (p < 0.001, F-test) and a borderline significant effect (p = 0.051, F-test) of treatment × time for *TaASN3*. At 7 days post-anthesis, the expression levels of the two genes were similar (Supplementary Table S5; Fig. 1b), but with sufficient nitrogen supplied the expression of *TaASN1* declined over time while that of *TaASN3* remained approximately constant, while under nitrogen-deficient conditions the expression of *TaASN1* remained approximately constant over time while *TaASN3* expression increased. This meant that *TaASN3* was the more highly expressed by 28 days post-anthesis, and both genes were more highly expressed under nitrogen deficiency than sufficiency by 28 days post-anthesis.

Finally, embryo and endosperm from grain at 14 days post-anthesis were separated and expression of the three asparagine synthetase genes was compared in the two tissues. The results showed that all three genes were more highly expressed (p < 0.05, t-tests) in the embryo than the endosperm (Fig. 1c, log_2_(1/NRQ) means: *TaASN1*: −0.516 (embryo), −2.025 (endosperm), p < 0.001; *TaASN2*: −4.178 (embryo), −2.228 (endosperm), p = 0.012; *TaASN3*: −2.453 (embryo), −1.628 (endosperm), p = 0.012), consistent with the higher concentrations of free asparagine that have been measured in the bran fractions of wheat grain compared with the white flour fractions (Shewry et al., 2009). *TaASN2* was the most highly expressed of the three genes in both tissues, followed by *TaASN3*, with very low but detectable expression of *TaASN1*. There was insufficient material from the other time-points to provide sufficient embryos for analysis, but this experiment showed that a larger experiment to investigate the differential expression of the genes in the embryo and endosperm was required.

This second, larger experiment was set up and as well as investigating expression of the *TaASN1*, *TaASN2* and *TaASN3* genes in the embryo, endosperm and other wheat tissues, it addressed how expression of the three genes responded to the supply of nitrogen in combination with sulphur. Variety Cadenza was used again, and the design of the experiment was a randomised block with three blocks of 16 pots. A factorial treatment structure was used consisting of two levels of nitrogen (N+, N−), two levels of sulphur (S+, S−) and four time points (14, 21, 28 and 35 days post-anthesis), making up the 16 treatment combinations per block. Also, from each pot, material was sampled for endosperm and embryo (from the developing grain), flag leaf, stem, and root. This gave 80 samples per block (five sub-samples per pot), making a total of 240 samples altogether. The same two reference genes were used: *Ta.2526.1.S1_at* and *TaActin*.

The qPCR data were processed to give results on the ct-scale and linear mixed modelling was applied to the data to test (F-tests) the main effects and interactions between the three factors of interest. Hence, the p-values for the effects of tissue, time (days post-anthesis), nitrogen (N+ versus N−) and sulphur (S+ versus S−), and their interactions, are given in Supplementary Table S6.

For *TaASN1* the full four-way interaction was significant (p < 0.001, F-test), so the full means table of log_2_(1/NRQ) values needed to be considered. For *TaASN2*, only the interaction between tissue and time factors was significant (p = 0.002, F-test), indicating that the changes in nutrients had no effect on the expression of this gene. The situation was more complex for *TaASN3*: there was a significant (p = 0.010, F-test) tissue by sulphur by time interaction, indicating that the effect of changing sulphur availability varied over time in the different tissues. There was also a tissue by nitrogen by sulphur interaction (p < 0.001, F-test), indicating an overall differential effect of nitrogen and sulphur on the different tissues. Finally, there was an interaction between nitrogen and time (p = 0.002, F-test), indicating an overall differential longitudinal effect of nitrogen.

**Fig. 2:**
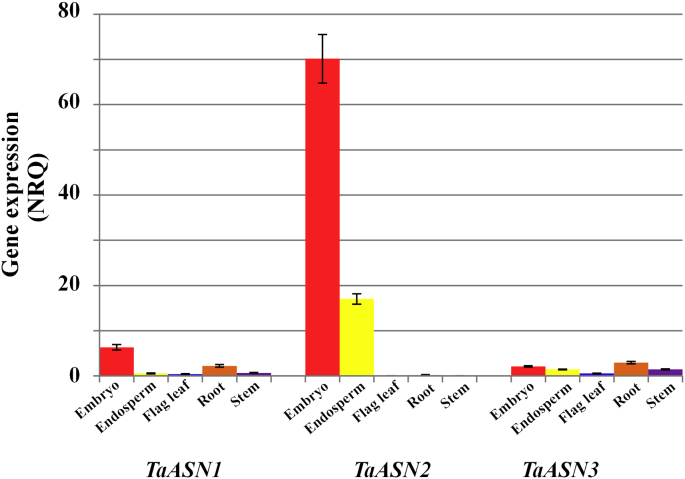
Differential expression of asparagine synthetase genes (*TaASN1*, *TaASN2* and *TaASN3*) in different tissues of wheat (*Triticum aestivum*) cv. Cadenza plants grown with nitrogen and sulphur supplied. Expression was measured at 21 days post-anthesis by real-time PCR and the graphs show the NRQ means and standard errors. Statistical analysis of the data is provided in Supplementary Tables S6–S9.

The relevant means are given in Supplementary Tables S7–S9 and the differential tissue-specific regulation of the three genes is illustrated in Fig. 2, which shows the NRQ means and standard errors for expression in different tissues at 21 days post-anthesis in plants fully supplied with both nitrogen and sulphur. The figure shows clearly that at this stage of development *TaASN2* expression in the embryo and endosperm represented by far the highest expression of any of the genes in any tissue, with expression of this gene in the embryo more than three times higher than that in the endosperm and at least 10 times higher than *TaASN1* or *TaASN3* expression in any tissue. On the other hand, expression of *TaASN2* in the flag leaf, root or stem was almost undetectable, while both *TaASN1* and *TaASN3* were expressed in all of these tissues, with *TaASN3* being expressed most highly in roots and *TaASN1* in the embryo.

**Fig. 3:**
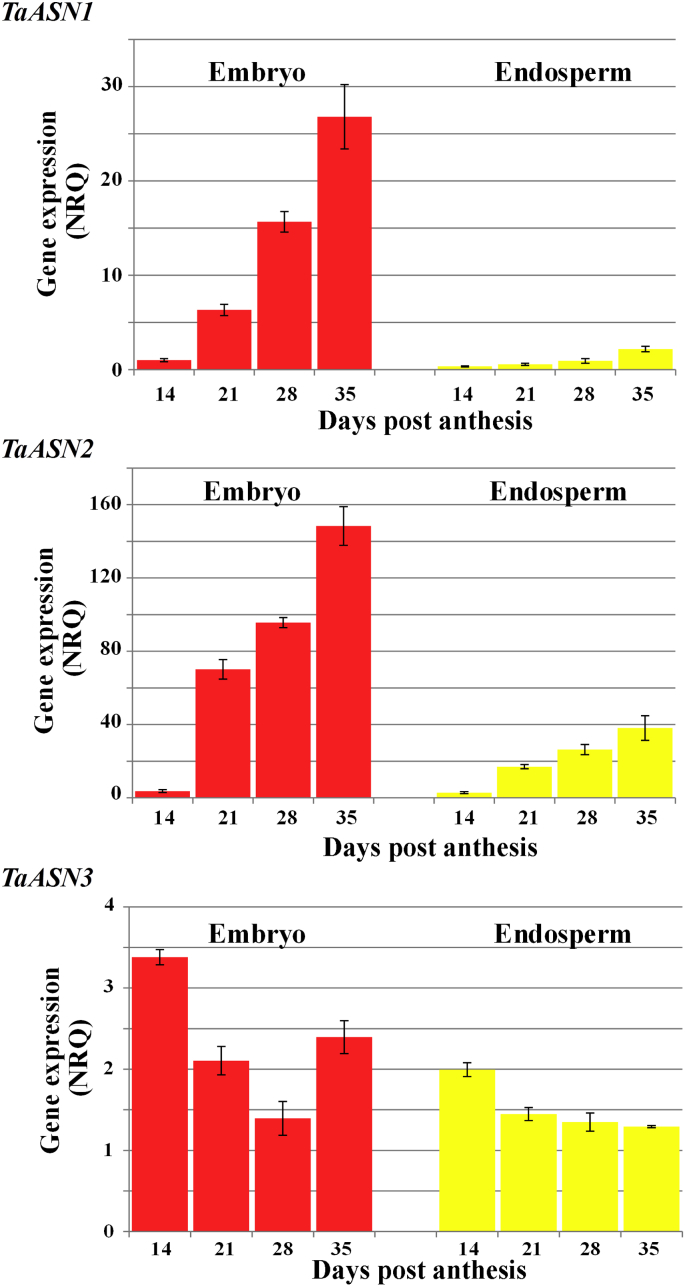
Expression of asparagine synthetase genes (*TaASN1*, *TaASN2* and *TaASN3*) in embryo and endosperm of wheat (*Triticum aestivum*) cv. Cadenza plants grown with nitrogen and sulphur supplied. Expression was measured at 14, 21, 28 and 35 days post-anthesis by real-time PCR and the graphs show the NRQ means and standard errors. Statistical analysis of the data is provided in Supplementary Tables S6–S9.

The three genes also showed differential expression through endosperm and embryo development in the situation of fully supplied nitrogen and sulphur, and this is illustrated in Fig. 3. Both *TaASN1* and *TaASN2* increased in expression in both tissues from 14 through 21 and 28 days–35 days post-anthesis. In plants fully supplied with both nitrogen and sulphur, expression of *TaASN1* increased approximately 6-fold in the endosperm in this period and 26-fold in the embryo, while *TaASN2* expression increased approximately 13- and 40-fold, respectively, in the two tissues. In contrast, expression of *TaASN3* was higher than that of *TaASN1* and comparable with that of *TaASN2* in both embryo and endosperm at 14 days post-anthesis but declined through development (although expression in the embryo recovered somewhat between 28 and 35 days post-anthesis), with the result that both *TaASN1* and particularly *TaASN2* were expressed many times more highly than *TaASN3* at the later stages of development.

**Fig. 4:**
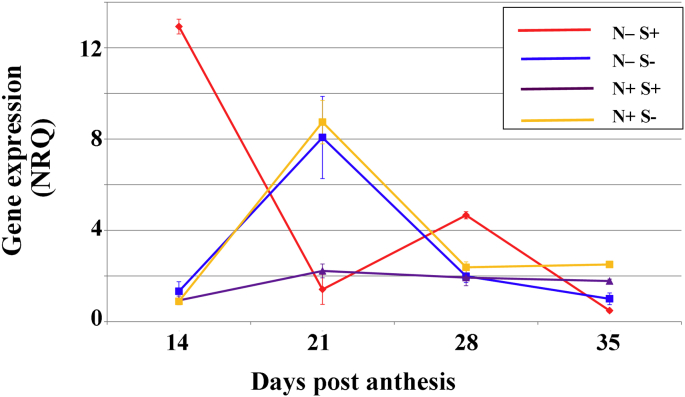
Expression of asparagine synthetase gene, *TaASN1*, in roots of wheat (*Triticum aestivum*) cv. Cadenza plants grown with different combinations of nitrogen and sulphur supply (N+ and S+) or deprivation (N− and S−). Expression was measured at 14, 21, 28 and 35 days post-anthesis by real-time PCR and the graphs plot the NRQ means and standard errors. Statistical analysis of the data is provided in Supplementary Tables S6 and S7.

The effects of nitrogen and sulphur supply on *TaASN1* gene expression in roots is shown in Fig. 4. *TaASN1* has previously been shown to increase in expression in response to sulphur deficiency in leaves of wheat seedlings (Byrne et al., 2012), but a more complicated picture emerged in these more mature plants, with nitrogen and sulphur interacting and having different effects on expression of the gene through development. Highest expression occurred in the high sulphur, low nitrogen roots at 14 days post-anthesis but this declined steeply so that this treatment gave the lowest expression at 21 days post-anthesis before rising and falling again at 28 and 35 days post-anthesis. Sulphur deprivation caused an increase in expression regardless of nitrogen supply but only at 21 days post-anthesis, while supply of sufficient nitrogen and sulphur resulted in low gene expression throughout.

### 3.3 Analysis of TaASN1, 2 and 3 gene expression in grain from sulphur-fed and -deprived field-grown plants

**Fig. 5:**
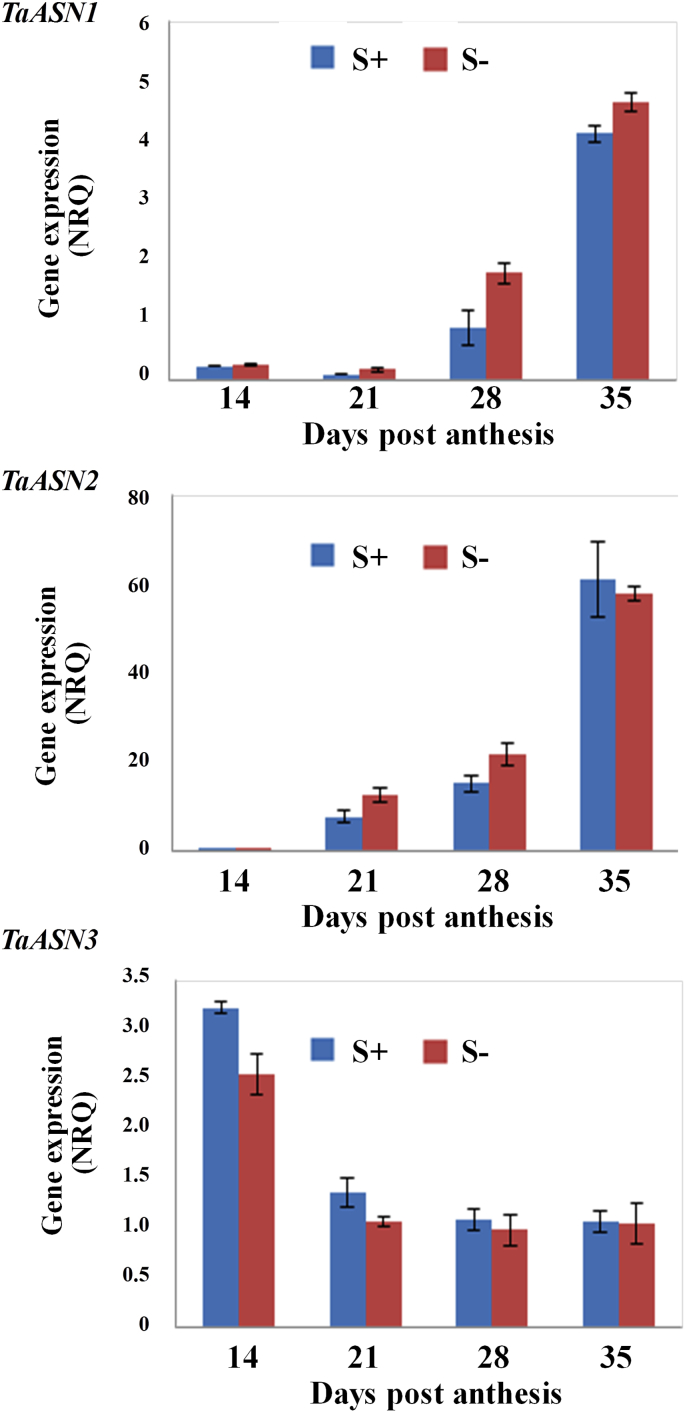
Expression of asparagine synthetase genes (*TaASN1*, *TaASN2* and *TaASN3*) in developing grains of wheat (*Triticum aestivum*) cv. Spark grown with or without sufficient sulphur in a field trial at the Rothamsted Research Woburn Farm in Bedfordshire, United Kingdom, in 2007–2008. Expression was measured at 14, 21, 28 and 35 days post-anthesis by real-time PCR and the graphs show the NRQ means and standard errors. Statistical analysis of the data is provided in Supplementary Tables S10 and S11.

The expression levels of *TaASN1*, *2* and *3* were compared in grain from wheat, cv. Spark that had been grown in a field trial in 2007–2008, with or without sulphur supplied. The trial had been conducted at Rothamsted Research's Woburn farm site, where the sandy loam soil is severely sulphur deficient (Riley et al., 2002). Total RNA from developing grain had been prepared after harvest at 14, 21, 28 and 35 days post-anthesis and stored at −80 °C. Expression of the three genes was determined by qPCR and the data were processed to give results on the ct-scale. Linear mixed modelling was then applied to test (F-tests) the main effects and interactions. The p-values for the effects of time (days post-anthesis), treatment (S+ versus S−), and the interaction between the two (treatment × time) are given in Supplementary Table S10, and the relevant means, SED (df) and LSD (5%) values for comparisons are given in Supplementary Table S11. The results are shown graphically in Fig. 5.

The patterns of gene expression matched those from the pot-grown plants very closely (Fig. 1, Fig. 3), although the big increase in expression of *TaASN1* and *TaASN2* that occurred between 14 and 21 days post-anthesis in the pot-grown plants occurred between 21 and 28 days post-anthesis in the field-grown plants. This probably reflected slower seed development in the field plants. In the field experiment, however, sulphur supply did have a significant effect on the expression of both *TaASN1* (p = 0.004, F-test) and *TaASN2* (p = 0.010, F-test). Notably, *TaASN1* gene expression almost doubled in response to sulphur deprivation at 21 days post-anthesis, and more than doubled at 28 days post-anthesis, in comparison to the corresponding supplied sulphur condition at those time points.

### 3.4 Identification of a putative regulatory motif in the promoter of TaASN1

The expression analyses performed in the study showed that *TaASN1* gene expression was responding to nitrogen and sulphur availability, and a previous study (Byrne et al., 2012) also found that *TaASN1* expression increased in the leaves of immature wheat plants in response to sulphur deficiency. The expression of *TaASN1* in the leaves of these immature plants, and its increase in response to sulphur deficiency, was repressed by over-expression of TaGCN2, a general control non-derepressible-2 (GCN2)-type protein kinase (Byrne et al., 2012). GCN2-type protein kinases phosphorylate the α subunit of translation initiation factor eIF2. This causes a general inhibition of protein synthesis, but in yeast (*Saccharomyces cereviseae*) it also brings about the translational up-regulation of a transcription factor, GCN4. Plants do not appear to have a homologue of GCN4, or at least not one that is recognisable on the basis of amino acid sequence similarity alone (Halford, 2006), but some plant genes do contain motifs that match the GCN4 binding site. In cereal storage protein genes, for example, including those encoding α, γ and ω gliadins and low molecular weight subunits in wheat, a GCN4-like motif, ATGAGTCAT, sometimes called the nitrogen element or N motif, forms part of the ‘prolamin box’ enhancer (reviewed by Shewry et al., 2003). The position of the prolamin box is highly conserved at approximately 250 base pairs upstream of the transcription start site and the GCN4-like motif binds a bZIP-type transcription factor called SPA (Albani et al., 1997).

In the light of this, the 5′ flanking regions of the asparagine synthetase genes were identified in wheat genome data (Wilkinson et al., 2012) and analysed for the presence of similar motifs. A perfect match for the N motif, ATGAGTCATC, was found in *TaASN1* but not *TaASN2* or *TaASN3*. Genomic data for *Aegilops tauschii*, barley and *Brachypodium distachyon* were also analysed and in each case *ASN1* genes were found to contain an identical motif in a similar position. The promoter nucleotide sequences are aligned in Supplementary Fig. S2, with the *T. aestivum*, *Ae. tauschii* and barley sequences showing >98% sequence identity with each other and approximately 58% identity with the *B. distachyon* sequence.

### 3.5 Identification of a fourth wheat asparagine synthetase gene, TaASN4, and phylogenetic analysis of the cereal asparagine synthetase gene family

**Fig. 6:**
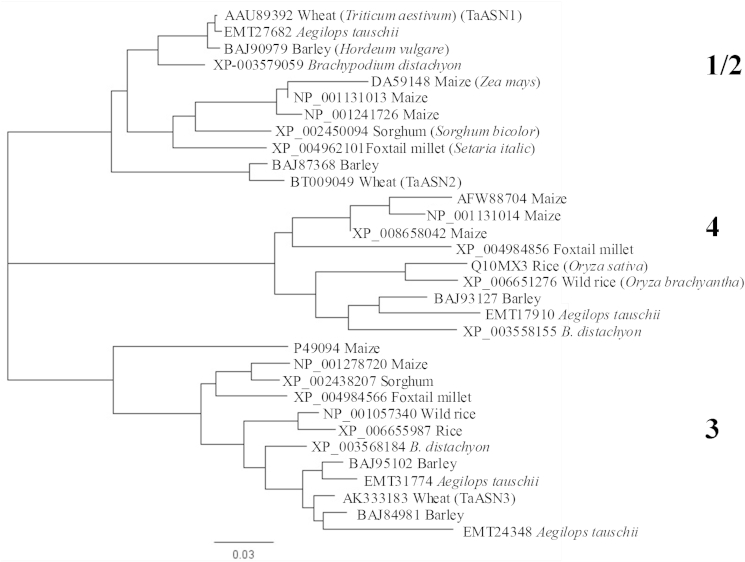
Phylogenetic analysis of cereal asparagine synthetases, generated using Geneious version 8 (Kearse et al., 2012). The sequences were identified in a BLAST search of the non-redundant protein database using *TaASN1* as the query sequence, with the ‘monocot’ filter, and the output was edited down to include only single representatives of groups of identical or almost identical proteins from the same species. The proteins are annotated by their database accession number and species. The three distinct clusters, 1/2, 3 and 4 are indicated.

A BLAST search of the monocot sequences in the non-redundant protein database was carried out using TaASN1 as the query sequence and the ‘monocot’ filter. The output was edited down to remove multiple representatives of groups of identical or almost identical proteins from the same species, in order to make the result easier to interpret. A phylogenetic analysis of the amino acid sequences of the proteins identified in this way was performed using the Geneious program (Kearse et al., 2012) and the output is shown in Fig. 6.

Currently there is no consistency in the annotation of different asparagine synthetases across groups of even closely related species such as these, with classification into numerically-defined groups (ASN1, 2, 3 etc.) being applied apparently on an *ad hoc* basis. The analysis performed here identified three clear groupings, with TaASN1 and 2 in one, TaASN3 in another and no bread wheat representative in the third. Since the expression analyses had shown that the genes encoding TaASN1 and TaASN2 were differentially regulated, the separate annotations were retained and this first group was called Group 1/2 (Fig. 6). To date Group 1/2 includes proteins from barley, *Ae. tauschii*, *B. distachyon*, maize, sorghum (*Sorghum bicolor*) and foxtail millet (*Setaria italica*), in addition to wheat. The group containing TaASN3 was called Group 3 (Fig. 6), and to date contains proteins from maize, foxtail millet, rice (*Oryza sativa*), wild rice (*Oryza brachyantha*), *B. distachyon*, barley and *Ae. tauschii* as well as wheat.

The other cluster was called Group 4 (Fig. 6), and currently contains proteins from cultivated and wild rice, *B. distachyon*, *Ae. tauschii*, foxtail millet and maize. While there were no representatives from bread wheat in this group (as of 14th January 2016), a BLAST search of wheat genome data (www.cerealsdb.uk.net; Wilkinson et al., 2012) did identify contigs containing sequences of nucleotides that were identical to the *Ae. Tauschii* gene sequence, although these were fragmented and could not be assembled into a single contig.

## 4 Discussion

Asparagine has long been considered to be an important nitrogen transport and storage molecule (Lea et al., 2007) and interest in the factors that control its synthesis, break-down and accumulation in crop plants has increased even further in recent years since it has been identified as a precursor for acrylamide, an important processing contaminant. Asparagine synthetase has already been the target of genetic interventions in potato, and a genetically modified potato variety with reduced asparagine synthetase gene expression in the tubers has been developed (Chawla et al., 2012) and was deregulated by the United States Department for Agriculture in 2014. The aim of the present study was to investigate the structure and expression of the asparagine synthetase gene family of wheat, setting the groundwork for similar genetic interventions.

Initially, three wheat asparagine synthetase genes were identified, but a fourth, hitherto uncharacterised gene, was identified from genome data at the end of the study. Phylogenetic analysis showed that all of the cereal asparagine synthetases identified to date fall into three clusters. The proteins that were annotated as TaASN1 and TaASN2 clustered together in the phylogenetic analysis, but because the expression analyses had shown the genes encoding these two proteins to be differentially regulated this cluster was annotated as Group 1/2, the others being Groups 3 and 4. This is consistent with the pattern reported for maize (Todd et al., 2008) and barley (Avila-Ospina et al., 2015).

The expression of *TaASN2* in the embryo and to a lesser extent the endosperm during mid to late grain development was by far the highest of any of the genes in any tissue. This suggests that most grain asparagine is likely to have been synthesised *in situ*, analagous to the situation in potato, in which asparagine accumulates to high concentrations in the tuber due to synthesis *in situ* rather than import from other parts of the plant (Muttucumaru et al., 2014). It suggests that genetic interventions should be targeted at *TaASN2*, and encourages optimism that such interventions could be made without compromising plant growth. However, it must be noted that the four asparagine synthases of maize are kinetically distinct with substantial differences in *K*_*m*_ (Gln) and *V*_*max*_ values (Duff et al., 2011), so gene expression levels do not tell the whole story.

Expression of both *TaASN1* and *TaASN2* increased through grain development, in both endosperm and embryo, continuing through to 35 days post-anthesis, when the grain is in its maturation phase. It is a reasonable hypothesis that high expression during maturation is not associated with protein synthesis but with accumulation of the free amino acid, perhaps for storage in preparation for germination. It is also notable that *TaASN1* expression has previously been shown to be induced by exogenous abscisic acid in wheat seedlings (Wang et al., 2005), and high concentrations of abscisic acid are also associated with the later stages of cereal grain development.

The problem of acrylamide formation in baked wheat products is exacerbated by the sensitivity of asparagine metabolism to nutrient supply (Curtis et al., 2009, Curtis et al., 2014b, Granvogl et al., 2007, Muttucumaru et al., 2006). It has been suggested that wheat uses free asparagine as a grain nitrogen store when deficiencies in other minerals, particularly sulphur, inhibit protein synthesis (Halford et al., 2012) and this is one example of how nutritional stress can affect crop composition (Halford et al., 2015). *TaASN1* has previously been shown to be up-regulated in the leaves of wheat seedlings in response to sulphur deprivation (Byrne et al., 2012), and in the present study both *TaASN1* and *TaASN2* expression increased in the grain during mid-development in field-grown plants in response to sulphur deprivation. However, only *TaASN1* responded to nitrogen or sulphur in the pot-based experiments, yet it did not show the clear response that had been reported for seedlings. Indeed, it showed a complex series of tissue-specific and developmentally-changing responses. The response of *TaASN1* gene expression to nutrient supply has been shown previously to be affected by over-expression of the protein kinase, TaGCN2 (Byrne et al., 2012), and it may involve a promoter element that is a perfect match for the so-called N-motif or GCN4-like motif (Shewry et al., 2003). This element was found in *TaASN1* genes from bread wheat, *Ae. tauschii*, barley and *B. distachyon*.

## References

[bib1] Albani D., Hammond-Kosack M.C.U., Smith C., Conlan S., Colot V., Holdsworth M., Bevan M.W. (1997). The wheat transcriptional activator SPA: a seed-specific bZIP protein that recognizes the GCN4-like motif in the bifactorial endosperm box of prolamin genes. Plant Cell.

[bib2] Avila-Ospina L., Marmagne A., Talbotec J., Krupinska K. (2015). The identification of new cytosolic glutamine synthetase and asparagine synthetase genes in barley (*Hordeum vulgare* L.), and their expression during leaf senescence. J. Exp. Bot..

[bib3] Byrne E.H., Prosser I., Muttucumaru N., Curtis T.Y., Wingler A., Powers S., Halford N.G. (2012). Over-expression of GCN2-type protein kinase in wheat has profound effects on free amino acid concentration and gene expression. Plant Biotechnol. J..

[bib4] Chawla R., Shakyra R., Rommens C.M. (2012). Tuber-specific silencing of asparagine synthetase-1 reduces the acrylamide-forming potential of potatoes grown in the field without affecting tuber shape and yield. Plant Biotechnol. J..

[bib5] Curtis T.Y., Muttucumaru N., Shewry P.R., Parry M.A., Powers S.J., Elmore J.S., Mottram D.S., Hook S., Halford N.G. (2009). Effects of genotype and environment on free amino acid levels in wheat grain: implications for acrylamide formation during processing. J. Agric. Food Chem..

[bib6] Curtis T.Y., Postles J., Halford N.G. (2014). Reducing the potential for processing contaminant formation in cereal products. J. Cereal Sci..

[bib7] Curtis T., Halford N.G., Powers S.J., McGrath S.P., Zazzeroni R. (2014). Effect of Sulphur Fertilisation on the Acrylamide-forming Potential of Wheat.

[bib8] Duff S.M.G., Qi Q., Reich T., Wu X., Brown T., Crowley J.H., Fabbri B. (2011). A kinetic comparison of asparagine synthetase isozymes from higher plants. Plant Physiol. Biochem..

[bib9] Granvogl M., Wieser H., Koehler P., Von Tucher S., Schieberle P. (2007). Influence of sulfur fertilization on the amounts of free amino acids in wheat. Correlation with baking properties as well as with 3-aminopropionamide and acrylamide generation during baking. J. Agric. Food Chem..

[bib10] Halford N.G. (2006). Regulation of carbon and amino acid metabolism: roles of sucrose nonfermenting-1-related protein kinase-1 and general control nonderepressible-2-related protein kinase. Adv. Bot. Res. Inc. Adv. Plant Pathol..

[bib11] Halford N.G., Curtis T.Y., Muttucumaru N., Postles J., Elmore J.S., Mottram D.S. (2012). The acrylamide problem: a plant and agronomic science issue. J. Exp. Bot..

[bib12] Halford N.G., Curtis T.Y., Chen Z., Huang J. (2015). Darwin review: effects of abiotic stress and crop management on cereal grain composition: implications for food quality and safety. J. Exp. Bot..

[bib13] Kearse M., Moir R., Wilson A., Stones-Havas S., Cheung M., Sturrock S., Buxton S., Cooper A., Markowitz S., Duran C., Thierer T., Ashton B., Mentjies P., Drummond A. (2012). Geneious basic: an integrated and extendable desktop software platform for the organization and analysis of sequence data. Bioinformatics.

[bib14] Lea P.J., Sodek L., Parry M.A., Shewry P.R., Halford N.G. (2007). Asparagine in plants. Ann. App. Biol..

[bib15] Martinek P., Klem K., Vanova M., Bartackova V., Vecerkova L., Bucher P., Hajslova J. (2009). Effects of nitrogen nutrition, fungicide treatment and wheat genotype on free asparagine and reducing sugars content as precursors of acrylamide formation in bread. Plant Soil Environ..

[bib16] Muttucumaru N., Halford N.G., Elmore J.S., Dodson A.T., Parry M., Shewry P.R., Mottram D.S. (2006). Formation of high levels of acrylamide during the processing of flour derived from sulfate-deprived wheat. J. Agric. Food Chem..

[bib17] Muttucumaru N., Keys A., Parry M.A.J., Powers S.J., Halford N.G. (2014). Photosynthetic assimilation of ^14^C into amino acids in potato (*Solanum tuberosum*) and asparagine in the tubers. Planta.

[bib18] Postles J., Powers S.J., Elmore J.S., Mottram D.S., Halford N.G. (2013). Effects of variety and nutrient availability on the acrylamide forming potential of rye grain. J. Cereal Sci..

[bib19] Ramakers C., Ruijter J., Lekanne-Deprez R., Moorman A. (2003). Assumption-free analysis of quantitative real-time polymerase chain reaction (PCR) data. Neurosci. Lett..

[bib20] Rieu I., Powers S.J. (2009). Real-time quantitative RT-PCR: design, calculations and statistics. Plant Cell.

[bib21] Riley N.G., Zhao F.J., McGrath S.P. (2002). Leaching losses of sulphur from different forms of sulphur fertilizers: a field lysimeter study. Soil Use Manag..

[bib22] Shewry P.R., Halford N.G., Lafiandra D. (2003). The genetics of wheat gluten proteins. Adv. Genet..

[bib23] Shewry P.R., Zhao F.-J., Gowa G.B., Hawkins N.D., Ward J.L., Beale M.H., Halford N.G., Parry M.A.J., Abécassis J. (2009). Sulphur nutrition differentially affects the distribution of asparagine in wheat grain. J. Cereal Sci..

[bib24] Todd J., Screen S., Crowley J., Penga J., Andersen A., Brown T., Qi Q., Fabri B., Duff S.M.G. (2008). Identification and characterization of four distinct asparagine synthetase (*AsnS*) genes in maize (*Zea mays* L.). Plant Sci..

[bib25] Tsai F.Y., Coruzzi G.M. (1990). Dark-induced and organ-specific expression of two asparagine synthetase genes in *Pisum sativum*. EMBO J..

[bib26] Untergasser A., Cutcutache I., Koressaar T., Ye J., Faircloth B.C., Remm M., Rozen S.G. (2012). Primer3-new capabilities and interfaces. Nucleic Acids Res..

[bib27] Verwoerd T.C., Dekker B.M.M., Hoekema A. (1989). A small-scale procedure for the rapid isolation of plant RNAs. Nucleic Acids Res..

[bib28] Wan Y., Shewry P.R., Hawkesford M.J. (2013). A novel family of γ-gliadin genes are highly regulated by nitrogen supply in developing wheat grain. J. Exp. Bot..

[bib29] Wang H., Liu D., Sun J., Zhang A. (2005). Asparagine synthetase gene *TaASN1* from wheat is up-regulated by salt stress, osmotic stress and ABA. J. Plant Physiol..

[bib30] Wilkinson P.A., Winfield M.O., Barker G.L.A., Allen A.M., Burridge A., Coghill J.A., Edwards K.J. (2012). CerealsDB 2.0: an integrated resource for plant breeders and scientists. BMC Bioinform..

[bib31] Winkler U., Schön W.J. (1980). Amino acid composition of the kernel proteins in barley resulting from nitrogen fertilization at different stages of development. J. Agron. Crop Sci..

