# Supplementary material for: Food safety: Structure and expression of the asparagine synthetase gene family of wheat
Source: J Cereal Sci. 2016 Mar;68:122–31. doi: 10.1016/j.jcs.2016.01.010 (PMC4829093; doi:10.1016/j.jcs.2016.01.010)
Supplement: Supplementary file 1 [file mmc1.docx]

**Food safety: Structure and expression of the asparagine synthetase gene family of wheat**

**Runhong Gao, Tanya Y. Curtis, Stephen J. Powers, Hongwei Xu, Jianhua Huang^b^, and Nigel G. Halford**

**Supplementary Data**

**Fig. S1.** Alignment of the amino acid sequences of wheat (*Triticum aestivum*) cv. Cadenza asparagine synthetases TaASN1, TaASN2 and TaASN3. Identical residues at the same position are highlighted in black, and similar residues at the same position (conservative substitutions) in grey. Conserved residues which have been shown to play essential roles in glutamine-dependent asparagine synthetase activity are indicated: black circles indicate essential residues of the glutamine-binding domain, asterisks indicate residues involved in ATP-anchoring, and black squares show residues involved in aspartate binding.

**
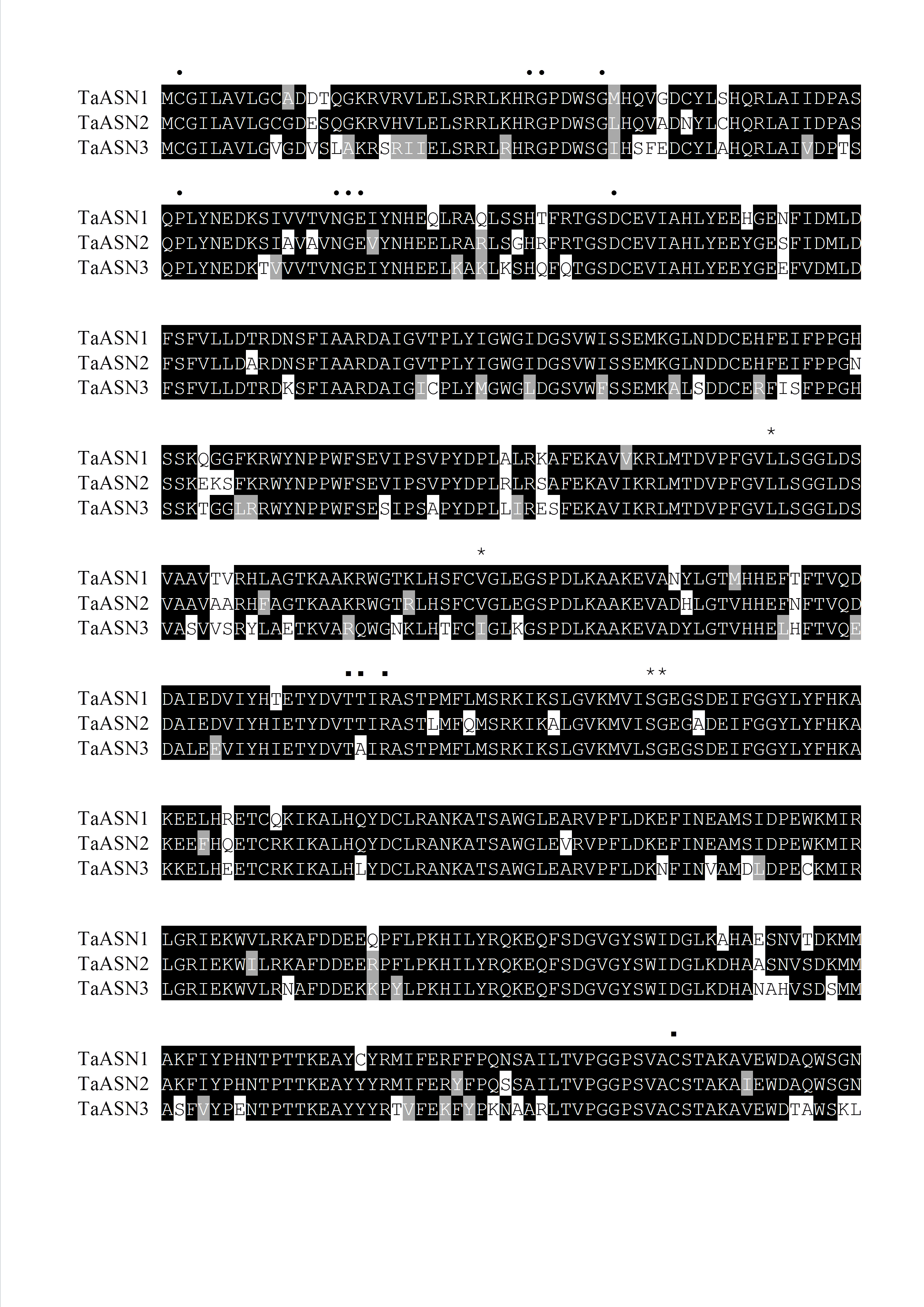
**

**Fig. S2.** Alignment of the nucleotide sequences of the promoter regions of *TaASN1* genes from bread wheat (*T. aestivum*), wild wheat (*Ae. tauschii*), barley (*H. vulgare*) and *Brachypodium distachyon*. Numbering is given for the *T. aestivum* gene, with respect to the translation start site, which is highlighted in purple. Putative TATA boxes are highlighted in blue and the putative N-motif (also known as the GCN4-like motif) is highlighted in red. Otherwise, identical bases at the same position are highlighted in black.

**
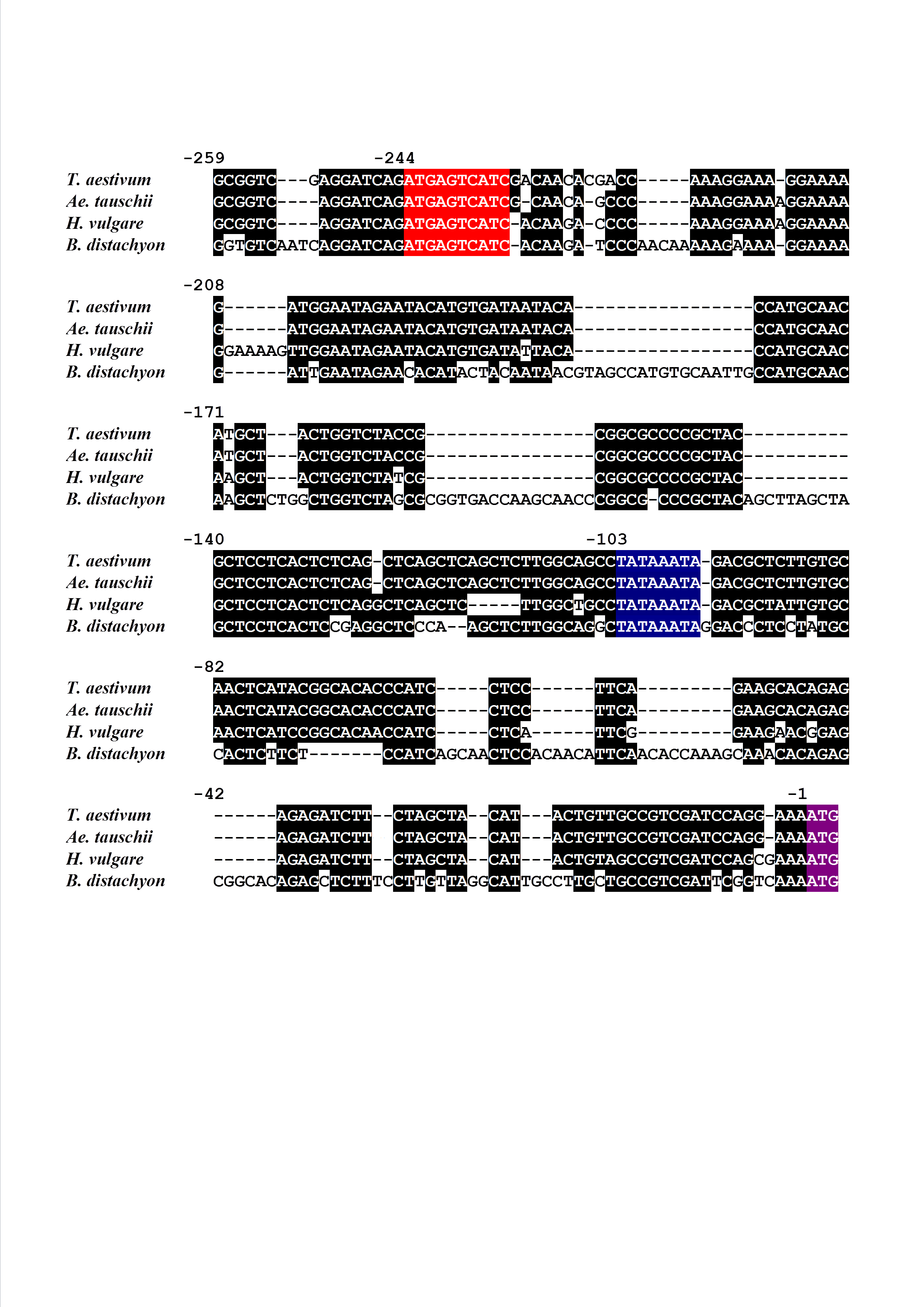
**

**Table S1. Oligonucleotide primers used for gene expression analysis by quantitative polymerase chain reaction**

| **Gene** | **5’ Primer** | **3’ Primer** |
| --- | --- | --- |
| *TaASN1* | 5’-GGCCTATGAACAGGAGCATCT | 5’-ACAGGACACCATCAACTCTCAAT |
| *TaASN2* | 5’-TGTGGGAGGTAGCAACAAGC | 5’-GCGATGCACACAACCGTTCT |
| *TaASN3* | 5’-AGAGCATCCTAGTCGAAACTGG | 5’-ACGGACAAGTTGCAAGGAAG |
| *Ta.2526.1.S1_at* | 5’- ACTGCAGCTGAGGAAAGAGC | 5’- CACACAGTGCCTGATCGAAG |
| *TaActin* | 5’-TTGCTGACCGTATGAGCAAG | 5’-ACCCTCCAATCCAGACACTG |

**Table S2.**

Linear mixed model results for expression of asparagine synthetase genes (*TaASN1*, *TaASN2* and *TaASN3*) in wheat (*Triticum aestivum*) cv. Cadenza developing seeds from plants grown with or without sufficient nitrogen. P-values in bold indicate the significant (p < 0.051, F-test) terms that were selected for inspection.

| **Gene** | **Treatment** | **Time** | **Treat × time** | ***s^2^*, residual df** |
| --- | --- | --- | --- | --- |
| *TaASN1* | <0.001 | <0.001 | **0.051** | 0.134, 13 |
| *TaASN2* | 0.598 | **<0.001** | 0.628 | 0.149, 10 |
| *TaASN3* | 0.540 | **<0.001** | 0.739 | 0.065, 14 |

**Table S3.**

Relevant means tables, on the log_2_ (1/NRQ) scale, for comparison of effect of treatment, time, and the interaction between the two (treat × time) for asparagine synthetase genes (*TaASN1*, *TaASN2* and *TaASN3*) in developing seeds of wheat (*Triticum aestivum*) cv. Cadenza grown with or without sufficient nitrogen. Note that, as for ct values, a low log_2_ (1/NRQ) indicates high gene expression, whereas a high log_2_ (1/NRQ) indicates low gene expression.

| **Gene** | **Treatment** | **Time (days post anthesis)** | | | | **df** | **LSD (5%)** |
| --- | --- | --- | --- | --- | --- | --- | --- |
| *TaASN1* |  | **7** | **14** | **21** | **28** |  |  |
|  | N+ | 0.790 | 1.653 | -1.014 | -2.735 | 13 | 0.647 |
|  | N- | 0.735 | 2.837 | 0.009 | -2.059 |  |  |
| *TaASN2* | | 7.324 | -2.085 | -4.698 | -5.632 | 10 | 0.4969 |
| *TaASN3* | | -2.393 | -1.355 | -0.817 | -0.660 | 14 | 0.3160 |

**Table S4**

Linear mixed model results for expression of asparagine synthetase genes (*TaASN1*, *TaASN2* and *TaASN3*) in flag leaves of wheat (*Triticum aestivum*) cv. Cadenza grown with or without sufficient nitrogen. P-values in bold indicate the significant (p < 0.051, F-test) terms that were selected for inspection

| **Gene** | **Treatment** | **Time** | **Treat × time** | ***s^2^*, residual df** |
| --- | --- | --- | --- | --- |
| *TaASN1* | 0.254 | 0.032 | **0.007** | 0.246, 8 |
| *TaASN3* | <0.001 | 0.092 | **0.051** | 0.086, 13 |

**Table S5**

Relevant means tables, on the log_2_ (1/NRQ) scale, to show the effects of treatment, time, and the interaction between the two (treat × time) for asparagine synthetase genes (*TaASN1*, *TaASN2* and *TaASN3*) in flag leaves of wheat (*Triticum aestivum*) cv. Cadenza developing seeds grown with or without sufficient nitrogen. Note that, as for ct values, a low log_2_ (1/NRQ) indicates high gene expression, whereas a high log_2_ (1/NRQ) indicates low gene expression.

| **Gene** | **Treatment** | **Time (days post anthesis)** | | | | **df** | **LSD (5%)** |
| --- | --- | --- | --- | --- | --- | --- | --- |
|  |  | **7** | **14** | **21** | **28** |  |  |
| *TaASN1* | N+ | 0.14 | 1.58 | 1.74 | 2.69 | 8 | 0.952 |
|  | N- | 1.54 | 1.07 | 1.33 | 1.19 |  |  |
| *TaASN3* | N+ | 0.025 | 0.164 | -0.044 | 0.383 | 13 | 0.517 |
|  | N- | -0.242 | -0.359 | -0.989 | -0.877 |  |  |

**Table S6.**

Results of fitting a linear mixed model to log_2_(1/NRQ) data for genes *TaASN1*, *TaASN2* and *TaASN3* to test for main effects and interactions between *Tissue* (endosperm versus embryo), *N*, *S* and *Time* (days post anthesis) factors. The p-values for the F-test of each fixed effect model term are given. The model terms of importance for further interpretation are given in bold.

| **Term** | ***TaASN1*** | ***TaASN2*** | ***TaASN3*** |
| --- | --- | --- | --- |
| *Tissue* | <0.001 | <0.001 | <0.001 |
| *N* | <0.001 | 0.220 | <0.001 |
| *S* | 0.122 | 0.689 | <0.001 |
| *Time* | <0.001 | <0.001 | <0.001 |
| *Tissue.N* | <0.001 | 0.503 | 0.217 |
| *Tissue.S* | <0.001 | 0.679 | 0.011 |
| *N.S* | 0.084 | 0.988 | <0.001 |
| *Tissue.Time* | <0.001 | **0.002** | <0.001 |
| *N.Time* | <0.001 | 0.807 | **0.002** |
| *S.Time* | <0.001 | 0.986 | 0.202 |
| *Tissue.N.S* | <0.001 | 0.301 | **<0.001** |
| *Tissue.N.Time* | <0.001 | 0.819 | 0.720 |
| *Tissue.S.Time* | <0.001 | 0.547 | **0.010** |
| *N.S.Time* | <0.001 | 0.520 | 0.570 |
| *Tissue.N.S.Time* | **<0.001** | 0.127 | 0.841 |

**Table S7**

Expression of asparagine synthetase gene *TaASN1* in different tissues of wheat (*Triticum aestivum*) cv. Cadenza grown with different combinations of nitrogen and sulphur supply.

1. Relevant means, on the log_2_ (1/NRQ) scale, to show the effects of treatment and time in different tissues.
2. Relevant NRQ means and standard errors in parenthesis.

**a**

|  | **Treatment** | | **Time (days post anthesis)** | | | |
| --- | --- | --- | --- | --- | --- | --- |
| **Tissue** | **Nitrogen** | **Sulphur** | **14** | **21** | **28** | **35** |
| Embryo | + | + | 0.020 | -2.711 | -3.966 | -4.726 |
|  | + | - | -0.440 | -2.844 | -3.807 | -4.323 |
|  | - | + | -0.115 | -2.809 | -3.872 | -4.302 |
|  | - | - | 0.031 | -2.437 | -3.443 | -4.642 |
| Endosperm | + | + | 1.551 | 0.891 | 0.088 | -1.123 |
|  | + | - | 1.428 | 0.962 | 0.800 | -0.410 |
|  | - | + | 1.058 | 0.884 | -0.189 | -2.669 |
|  | - | - | 1.138 | 0.695 | 0.336 | -2.983 |
| Flag leaf | + | + | 1.199 | 1.394 | 1.220 | 1.933 |
|  | + | - | 1.342 | 2.640 | 2.191 | 2.410 |
|  | - | + | -0.709 | 1.256 | 1.463 | 0.751 |
|  | - | - | 1.100 | 0.883 | 1.657 | 0.957 |
| Root | + | + | 0.153 | -1.080 | -0.977 | -0.854 |
|  | + | - | 0.126 | -3.168 | -1.170 | -1.418 |
|  | - | + | -3.776 | -0.375 | -2.244 | 1.014 |
|  | - | - | -0.257 | -2.993 | -0.895 | 0.109 |
| Stem | + | + | 0.272 | 0.585 | 0.361 | -1.148 |
|  | + | - | 0.150 | -2.032 | 0.468 | -0.163 |
|  | - | + | -2.210 | 0.628 | 0.188 | 0.019 |
|  | - | - | 0.567 | -0.044 | -0.248 | -0.357 |

Average SED = 0.3433 on 119 df, Average LSD (5%) = 0.6797

b

|  | **Treatment** | | **Time (days post anthesis)** | | | |
| --- | --- | --- | --- | --- | --- | --- |
| **Tissue** | **N** | **S** | **14** | **21** | **28** | **35** |
| Embryo | **+** | **+** | 1.008 (0.163) | 6.332 (0.595) | 15.666 (1.090) | 26.792 (3.400) |
|  | **+** | **-** | 1.422 (0.150) | 7.195 (0.257) | 14.303 (2.490) | 20.191 (0.409) |
|  | **-** | **+** | 1.084 (0.040) | 7.123 (0.894) | 15.131 (2.688) | 19.717 (1.129) |
|  | **-** | **-** | 0.986 (0.147) | 5.342 (0.125) | 11.471 (0.873) | 25.462 (3.560) |
| Endosperm | **+** | **+** | 0.348 (0.054) | 0.560 (0.115) | 0.930 (0.238) | 2.188 (0.295) |
|  | **+** | **-** | 0.362 (0.013) | 0.512 (0.062) | 0.585 (0.057) | 1.298 (0.155) |
|  | **-** | **+** | 0.480 (0.045) | 0.567 (0.115) | 1.178 (0.048) | 6.694 (0.661) |
|  | **-** | **-** | 0.462 (0.046) | 0.639 (0.120) | 0.830 (0.157) | 8.295 (1.033) |
| Flag Leaf | **+** | **+** | 0.443 (0.072) | 0.398 (0.073) | 0.460 (0.108) | 0.258 (0.039) |
|  | **+** | **-** | 0.413 (0.090) | 0.158 (0.013) | 0.222 (0.019) | 0.192 (0.017) |
|  | **-** | **+** | 1.693 (0.555) | 0.430 (0.068) | 0.376 (0.076) | 0.598 (0.147) |
|  | **-** | **-** | 0.483 (0.113) | 0.545 (0.040) | 0.320 (0.022) | 0.549 (0.112) |
| Root | **+** | **+** | 0.933 (0.190) | 2.221 (0.302) | 1.930 (0.356) | 1.778 (0.083) |
|  | **+** | **-** | 0.888 (0.058) | 8.743 (0.953) | 2.379 (0.240) | 2.578 (0.088) |
|  | **-** | **+** | 12.929 (0.322) | 1.411 (0.654) | 4.661 (0.158) | 0.481 (0.073) |
|  | **-** | **-** | 1.329 (0.419) | 8.068 (1.800) | 1.990 (0.276) | 0.999 (0.258) |
| Stem | **+** | **+** | 0.908 (0.306) | 0.673 (0.073) | 0.759 (0.100) | 2.221 (0.345) |
|  | **+** | **-** | 0.894 (0.041) | 4.086 (0.608) | 0.772 (0.212) | 1.152 (0.134) |
|  | **-** | **+** | 4.609 (0.158) | 0.652 (0.041) | 0.911 (0.183) | 0.961 (0.079) |
|  | **-** | **-** | 0.681 (0.082) | 1.063 (0.186) | 1.307 (0.413) | 1.345 (0.268) |

**Table S8**

Expression of asparagine synthetase gene *TaASN2* in different tissues of wheat (*Triticum aestivum*) cv. Cadenza grown with different combinations of nitrogen and sulphur supply.

1. Relevant means, on the log_2_ (1/NRQ) scale, to show the effects of time and tissue type.
2. Relevant NRQ means and standard errors in parenthesis.

**a**

|  | **Time (days post anthesis)** | | | |
| --- | --- | --- | --- | --- |
| **Tissue** | **14** | **21** | **28** | **35** |
| Embryo | -1.618 | -6.136 | -6.586 | -7.209 |
| Endosperm | -1.209 | -4.093 | -4.612 | -5.021 |
| Flag Leaf | 10.239 | 10.431 | 10.657 | 9.925 |
| Root | 9.142 | 6.297 | 5.681 | 8.584 |
| Stem | 11.252 | 12.225 | 10.154 | 9.432 |

Average SED = 1.1180 on 137 df, Average LSD (5%) = 2.2102

**b**

| **Tissue** | **Time (days post anthesis)** | | | |
| --- | --- | --- | --- | --- |
|  | **14** | **21** | **28** | **35** |
| **Embryo** | 3.65 (0.78) | 70.13 (5.38) | 95.58 (2.76) | 148.36 (10.56) |
| **Endosperm** | 2.83 (0.57) | 17.02 (1.13) | 26.37 (3.22) | 38.07 (6.72) |
| **Flag Leaf** | 0.00 (0.00) | 0.00 (0.00) | 0.00 (0.00) | 0.00 (0.00) |
| **Root** | 0.01 (0.00) | 0.16 (0.11) | 0.02 (0.00) | 0.02 (0.01) |
| **Stem** | 0.00 (0.00) | 0.00 (0.00) | 0.00 (0.00) | 0.01 (0.00) |

**Table S9**

Expression of asparagine synthetase gene *TaASN3* in different tissues of wheat (*Triticum aestivum*) cv. Cadenza grown with different combinations of nitrogen and sulphur supply.

1. Relevant means, on the log_2_ (1/NRQ) scale, to show the effects of sulphur × time in different tissues.
2. Relevant means, on the log_2_ (1/NRQ) scale, to show the effects of tissue type × nitrogen supply × sulphur supply.
3. Relevant means, on the log_2_ (1/NRQ) scale, to show the effect of nitrogen × time.
4. Relevant NRQ means and standard errors in parenthesis for sulphur × time in different tissues.
5. Relevant NRQ means and standard errors in parenthesis for tissue type × nitrogen supply × sulphur supply.
6. Relevant NRQ means and standard errors in parenthesis for nitrogen × time.

**a**

|  |  | **Time (days post anthesis)** | | | |
| --- | --- | --- | --- | --- | --- |
| **Tissue** | **Sulphur** | **14** | **21** | **28** | **35** |
| Embryo | + | -1.7536 | -1.0494 | -0.4029 | -1.2312 |
|  | - | -1.5853 | -1.1843 | -0.4132 | -1.0062 |
| Endosperm | + | -0.9891 | -0.5223 | -0.4056 | -0.3498 |
|  | - | -1.0655 | -0.9349 | -0.4408 | -0.8467 |
| Flag Leaf | + | 0.8197 | 0.8617 | 0.5599 | 0.3671 |
|  | - | 0.5696 | 0.3184 | 0.5693 | 0.4376 |
| Root | + | -1.4425 | -1.5345 | -1.5719 | -1.9600 |
|  | - | -1.4307 | -1.4424 | -1.8839 | -1.8360 |
| Stem | + | -0.6631 | -0.5088 | -0.4457 | -1.2206 |
|  | - | -0.6721 | -0.7032 | -0.8118 | -1.6892 |

Average SED = 0.1498 on 118 df, Average LSD (5%) = 0.29660

**b**

|  |  | **Sulphur** | |
| --- | --- | --- | --- |
| **Tissue** | **Nitrogen** | **+** | **-** |
| Embryo | + | -0.9319 | -1.0184 |
|  | - | -1.2867 | -1.0760 |
| Endosperm | + | -0.5846 | -0.6776 |
|  | - | -0.5488 | -0.9663 |
| Flag Leaf | + | 0.5079 | 0.6554 |
|  | - | 0.7963 | 0.2920 |
| Root | + | -1.6700 | -1.4492 |
|  | - | -1.5845 | -1.8473 |
| Stem | + | -0.6553 | -0.6972 |
|  | - | -0.7638 | -1.2409 |

Average SED = 0.1058 on 118 df, Average LSD (5%) = 0.2095

**c**

|  | **Time (days post anthesis)** | | | |
| --- | --- | --- | --- | --- |
| **Nitrogen** | **14** | **21** | **28** | **35** |
| + | -0.8269 | -0.6199 | -0.4379 | -0.7236 |
| - | -0.8156 | -0.7200 | -0.6114 | -1.1434 |

Average SED = 0.07140 on 28 df, Average LSD (5%) = 0.14623

**d**

| **Tissue** | **S** | **Time** | | | |
| --- | --- | --- | --- | --- | --- |
|  |  | **14** | **21** | **28** | **35** |
| **Embryo** | **S+** | 3.379 (0.094) | 2.105 (0.176) | 1.394 (0.209) | 2.395 (0.202) |
|  | **S-** | 3.005 (0.072) | 2.277 (0.068) | 1.361 (0.125) | 2.028 (0.128) |
| **Endosperm** | **S+** | 1.994 (0.085) | 1.447 (0.079) | 1.348 (0.112) | 1.293 (0.114) |
|  | **S-** | 2.119 (0.152) | 1.914 (0.040) | 1.383 (0.093) | 1.898 (0.269) |
| **Flag Leaf** | **S+** | 0.591 (0.074) | 0.566 (0.059) | 0.696 (0.070) | 0.781 (0.034) |
|  | **S-** | 0.689 (0.048) | 0.812 (0.055) | 0.696 (0.076) | 0.725 (0.080) |
| **Root** | **S+** | 2.743 (0.166) | 2.952 (0.258) | 3.020 (0.287) | 3.999 (0.426) |
|  | **S-** | 2.696 (0.142) | 2.777 (0.242) | 3.715 (0.194) | 3.658 (0.355) |
| **Stem** | **S+** | 1.587 (0.046) | 1.442 (0.110) | 1.374 (0.081) | 2.358 (0.263) |
|  | **S-** | 1.634 (0.173) | 1.675 (0.190) | 1.775 (0.122) | 3.196 (0.536) |

**e**

| **Tissue** | **N** | **S** | |
| --- | --- | --- | --- |
|  |  | **S+** | **S-** |
| **Embryo** | **N+** | 2.072 (0.244) | 2.118 (0.188) |
|  | **N-** | 2.564 (0.224) | 2.218 (0.191) |
| **Endosperm** | **N+** | 1.592 (0.137) | 1.672 (0.112) |
|  | **N-** | 1.490 (0.072) | 2.009 (0.141) |
| **Flag Leaf** | **N+** | 0.719 (0.043) | 0.645 (0.035) |
|  | **N-** | 0.582 (0.047) | 0.838 (0.038) |
| **Root** | **N+** | 3.285 (0.272) | 2.803 (0.157) |
|  | **N-** | 3.077 (0.223) | 3.713 (0.202) |
| **Stem** | **N+** | 1.569 (0.101) | 1.668 (0.125) |
|  | **N-** | 1.690 (0.165) | 2.406 (0.328) |

**f**

| **N** | **Time** | | | |
| --- | --- | --- | --- | --- |
|  | **14** | **21** | **28** | **35** |
| **N-** | 2.004 (0.171) | 1.706 (0.133) | 1.599 (0.181) | 1.944 (0.212) |
| **N+** | 2.086 (0.181) | 1.888 (0.163) | 1.717 (0.184) | 2.703 (0.259) |

**Table S10**

Linear mixed model results for expression of asparagine synthetase genes (*TaASN1*, *TaASN2* and *TaASN3*) in developing grains of wheat (*Triticum aestivum*) cv. Spark grown with or without sufficient sulphur in a field trial at the Rothamsted Research Woburn Farm in Bedfordshire, United Kingdom, in 2007-2008. P-values in bold indicate the significant (p < 0.05, F-test) terms that were selected for inspection

| **Gene** | **Treatment (Sulphur)** | **Time (Days post anthesis)** | **Treat × time** | ***s^2^*, residual df** |
| --- | --- | --- | --- | --- |
| *TaASN1* | **0.004** | **<0.001** | 0.100 | 0.1624, 13 |
| *TaASN2* | **0.010** | **<0.001** | 0.289 | 0.1103, 11 |
| *TaASN3* | 0.079 | **<0.001** | 0.787 | 0.0851, 14 |

**Table S11**

Relevant means, on the log_2_ (1/NRQ) scale, for expression of asparagine synthetase genes (*TaASN1*, *TaASN2* and *TaASN3*) in developing grains of wheat (*Triticum aestivum*) cv. Spark, grown with or without sufficient sulphur in a field trial at the Rothamsted Research Woburn Farm in Bedfordshire, United Kingdom, in 2007-2008, to show the significant effects of a. Time and b. Treatment, using the LSD (5%) values

**a**

| **Gene** | **Time (days post anthesis)** | | | | **df** | **LSD (5%)** |
| --- | --- | --- | --- | --- | --- | --- |
|  | **7** | **14** | **21** | **28** |  |  |
| *TaASN1* | 2.119 | 3.132 | -0.235 | -2.109 | 13 | 0.5026 |
| *TaASN2* | 6.029 | -3.241 | -4.158 | -5.863 | 11 | 0.4220 |
| *TaASN3* | -1.520 | -0.263 | -0.027 | -0.046 | 14 | 0.3612 |

**b**

| **Gene** | **Treatment** | | **df** | **LSD (5%)** |
| --- | --- | --- | --- | --- |
|  | **S+** | **S-** |  |  |
| *TaASN1* | 1.016 | 0.437 | 13 | 0.3354 |
| *TaASN2* | -1.597 | -2.019 | 11 | 0.2984 |
| *TaASN3* | -0.577 | -0.351 | 14 | 0.3612 |
